# Attention-deficit/hyperactivity disorder from preschool to school age: change and stability of parent and teacher reports

**DOI:** 10.1007/s00787-022-02019-1

**Published:** 2022-06-23

**Authors:** Kristin Romvig Overgaard, Beate Oerbeck, Svein Friis, Are Hugo Pripp, Heidi Aase, Guido Biele, Christine Baalsrud Ingeborgrud, Guilherme V. Polanczyk, Pål Zeiner

**Affiliations:** 1 Division of Mental Health and Addiction, Oslo University Hospital, Nydalen, P.B. 4959, 0424 Oslo, Norway; 2 Institute of Clinical Medicine, University of Oslo, Oslo, Norway; 3 Oslo Centre of Biostatistics and Epidemiology, Oslo University Hospital, Oslo, Norway; 4 Department of Child Health and Development, Norwegian Institute of Public Health, Oslo, Norway; 5 Department of Psychiatry, Faculdade de Medicina FMUSP, Universidade de Sao Paulo, Sao Paulo, Brazil

**Keywords:** ADHD, Children, Preschool, Diagnosis

## Abstract

Identifying attention-deficit/hyperactivity disorder (ADHD) in pre-schoolers may improve their development if treated, but it is unclear whether ADHD symptoms from this age are stable enough to merit treatment. We aimed to investigate the stability of parent- and teacher-reported ADHD symptoms and ADHD classified above the diagnostic symptom thresholds, including for hyperactivity-impulsivity (HI), inattention and combined presentations from age 3 to 8 years. This study is part of the longitudinal, population-based Norwegian Mother, Father and Child Cohort Study. At child age 3 years, parents were interviewed and teachers rated ADHD symptoms. At age 8 years, parents (*n* = 783) and teachers (*n* = 335) reported ADHD symptoms by the Child Symptom Inventory-4. We found a significant reduction in the mean number of parent-reported ADHD and HI symptoms from age 3 to 8 years, but otherwise similar mean numbers. Parent-reported ADHD symptoms were moderately correlated between ages, while correlations were low for teachers. A total of 77/108 (71%) of the children classified with parent-reported HI presentation at age 3 years were no longer classified within any ADHD presentation at age 8 years, the only clear trend across time for either informant. There was a low to moderate parent–teacher-agreement in the number of reported symptoms, and very low informant agreement for the classified ADHD presentations. Overall, clinicians should exercise caution in communicating concern about HI symptoms in preschool children. Age 3 years may be too early to apply the ADHD diagnostic symptom criteria, especially if parents and teachers are required to agree.

**Supplementary Information:**

The online version contains supplementary material available at 10.1007/s00787-022-02019-1.

## Introduction

The fifth edition of the Diagnostic and Statistical Manual of Mental Disorders (DSM-5) defines Attention-deficit/hyperactivity disorder (ADHD) by at least six out of nine symptoms of hyperactivity/impulsivity (HI) and/or inattention (IA), present in two or more settings [1]. The preschool ADHD prevalence rate is estimated to 1.9–3.3% [2–4], but most children are diagnosed in school age [5]. Nearly two decades ago, a review outlined some reasons why few pre-schoolers were diagnosed [6], reasons still likely to be influential in clinical practices today. First, it is a common belief that most pre-schoolers are highly active, making it challenging to identify ADHD at this age. Second, pre-schoolers develop rapidly (in cognitive-, motor- and social skills), complicating the diagnostic assessments. Third, it is often assumed that pre-schoolers will outgrow their problems and early diagnosis could therefore be potentially harmful.

Obtaining information from both parents and teachers is recommended to ascertain that the ADHD diagnostic thresholds are present in at least two settings [7]. Still, there will regularly be concern for children below these thresholds from either or both informants, particularly during preschool years when interventions are recommended to improve developmental trajectories [8]. Thus, a dimensional approach focusing on symptoms, could perhaps reach more pre-schoolers, potentially providing them with health services.

Reporting symptom-levels across time, one clinical study (*n* = 118) administrating a diagnostic interview at seven timepoints between ages 4–6 and 11–13 years, found an overall significant decline of HI symptoms, but stable IA symptoms over time [9]. Also, the preschool ADHD treatment study (PATS) (*n* = 207), including children diagnosed with ADHD at mean age 4.4 years with three follow-ups (to mean age 10.4 years), found that parent and teacher ADHD symptom scores decreased, but stayed moderate to severe [10]. Yet another study of 4–5-year-olds (*n* = 144) with annual follow-ups, reported HI to be common and IA to be rare at this age, with a reduction in HI and an increase in IA symptoms to age 6–7 years [11].

Reporting on the predictive power of symptoms from preschool onwards, a community study (*n* = 1042) following children every second year from age 4 to 10 years with repeated diagnostic parent interviews, found that the number of ADHD symptoms significantly correlated between timepoints (*r* = 0.32–0.62) [12], but did not include teacher reports. Another community study (*n* = 541), also using only parent reports (by diagnostic interviews) at three timepoints, reported that an increase by one ADHD-symptom at age 3 years gave a relative increased risk of 1.12 and 1.14 for an additional ADHD symptom at age 9 and 12 years, respectively [13]. Finally, one German community study (*n* = 477), with both parent and teacher questionnaire data, followed children three times from about age 5 to 7 years. That study reported high stability in the number of ADHD symptoms for the whole period, with moderate intercorrelations between two adjacent measurement points, calculated separately for parents and teachers [14]. Although these findings of moderate stability of ADHD symptoms from a young age onwards are promising, it may be difficult to transfer this approach to clinical practice where categorical diagnoses are often used.

Using a categorical approach to estimate diagnostic stability, previous clinical studies beginning before school age have reported a stability of ADHD diagnoses of ≥ 70% at follow-up 3–7 years later [9, 10, 15]. Community studies have been less convincing, with the above-mentioned German study reporting low to moderate stability of parent and teacher questionnaire-rated ADHD categories over time [14]. Another community study reported that ADHD identified by a diagnostic interview at age 3 years gave significantly increased risk of ADHD being diagnosed again at age 6 (odds ratio (OR) = 17.96) [13], 9 (OR = 5.63) and 12 years (OR = 6.80) [13]. However, that study was limited by very few children with ADHD diagnosis at the first assessment (*n* = 11), and by not including teacher reports.

Studies have also demonstrated ADHD as a dynamic disorder, with changes in ADHD presentations over time, rather than a stable category [9, 16]. In a study of pre-schoolers referred with externalizing problems, 37% (94/251) were classified with ≥ 6 HI symptoms at baseline (mean age 55 months) of whom 59% (55/94) were identified again with HI above threshold 18 months later. However, a smaller proportion (18%) were above the HI threshold at both 9- and 18-months follow-ups. Also, the authors noted new cases with time in 11% of the children, further underlining the instability of the HI presentation [16].

Among the above-mentioned studies beginning before school age, few have included teacher reports and could therefore not address the impact of different informants, recommended when diagnosing ADHD [1]. Low agreement between parent and teacher ratings, has long been recognized [17]. The PATS study reported low parent–teacher agreement of both HI and IA symptoms at mean age 4.4 years [18]. Similarly, one study with hyperactive-impulsive children (*n* = 104), found no significant parent–teacher agreement between ADHD symptoms or categorized ADHD (≥ 90th percentile) on a rating scale at baseline (mean age 4.4 years), and low agreement at age 6 years [19]. The above-mentioned German study (*n* = 392) found low to moderate ADHD symptom agreement across time between parents and teachers, and only one child was categorized with ADHD by both informants across all time points [14]. In our previous follow-up study from age 3 to 5 years (*n* = 957), we identified a small group (*n* = 20) of 3-year-old children who fulfilled ADHD criteria according to both parent and teacher reports. These children had high probabilities of being categorized with elevated ADHD symptoms at age 5 years [20].

In the present study, we followed a large cohort from preschool to school age, with the aim to investigate the stability of parent- and teacher-reported ADHD symptoms and ADHD classified above the diagnostic symptom thresholds during this time. Based on the literature presented above, we hypothesized that from age 3 to 8 years: (1) The mean level of parent- and teacher-reported HI symptoms would decrease, and IA symptoms would be stable or increase; (2) parent- and teacher-reported preschool ADHD symptoms would predict ADHD symptoms reported by the respective informants at age 8 years; (3) The proportions of categorized ADHD would have moderate stability, with low stability of the ADHD presentations according to both informants and (4**)** There would be low agreement between informants.

## Methods

### Participants

The Norwegian Mother, Father and Child Cohort Study (MoBa) is a population-based cohort study conducted by the Norwegian Institute of Public Health. Pregnant women were recruited from all over Norway from 1999 to 2008 (41% participation rate) [21]. The mothers were predominantly white Caucasians, recruited from Norway from 1999 to 2008 [22]. The current paper is from a clinical sub study on ADHD previously described [23, 24]. In this study, we oversampled children at risk for ADHD using the child age 3 year MoBa questionnaire with 11 items about ADHD; six items from the Child Behavior Checklist/1.5–5 (Cannot concentrate, Cannot sit still, Cannot stand waiting, Demands must be met immediately, Gets into everything, Quickly shifts activities) [25], and five items from the revised fourth edition of the DSM (DSM-IV-TR) (Easily distracted, Difficulty waiting his/her turn, Difficulty sustaining attention, Talks excessively, Does not seem to listen) [26]. Most of the invited participants (*n* = 2798) had scores ≥ 90th percentile on these 11 items, along with randomly selected children (*n* = 654). Thirty-five percent agreed to participate, and 1195 children took part in a 1-day clinical assessment including a diagnostic interview with parents (almost all mothers) from 2007 to 2011. In Norway, 95% of 3-year-olds attend preschool. When the children were age 3.5 years, their parents received the questionnaires by mail and gave the teachers their questionnaires and they mailed their responses to the study administrator. By the 8-year follow-up, 14 mothers withdrew from the MoBa-study, leaving 1181 participants. At age 8 years, about 66% (*n* = 783) of parents and 58% of teachers (*n* = 680) responded to the Child Symptom Inventory-4 (CSI-4).

## Measures

### Background variables

Birth date and child sex were obtained from the Norwegian Medical Birth Registry.

Length of parental education was obtained at the first MoBa assessment with questionnaires to mothers (about Week 17 of pregnancy).

### At 3 years of age

#### Parents’ classifications—the preschool age psychiatric assessment (PAPA)

The PAPA interview [27] was developed for children from age 2 to 5 years. The interviewer asks questions until s/he can decide whether the symptoms described meet the definitions provided in a glossary. A PAPA test–retest reliability study reported a test–retest intraclass correlation of 0.80 for classified ADHD [3]. In the present study, only ADHD symptoms persisting for ≥ 3 months were counted as present.

In line with our earlier studies [23, 24], we used information from the PAPA, and defined ADHD by the DSM-IV-TR criteria [26]. We reported number of ADHD symptoms and classified the ADHD presentations by the presence of at least six out of nine symptoms of HI and/or IA.

Oppositional defiant disorder (ODD) was defined by the presence of ≥ 4 DSM-IV ODD symptoms according to PAPA. Blinded to the questionnaire ratings, trained graduate psychology students conducted the interviews supervised by specialists in child psychology/psychiatry. A second blind rater rescored audiotapes of 79 randomly selected interviews, and the intraclass correlations were 0.97 and 0.99 for HI and IA symptoms, respectively, and 0.98 for ODD.

#### Teachers’ classifications—the Early Child Inventory-4 (ECI-4)

The ECI-4 was translated into Norwegian and back-translated into English by professional translators, with 108 items corresponding to the symptom lists of the child psychiatric disorders in the DSM-IV [28]. We used only the nine-item HI and IA subscales rated on a four-point Likert scale (never, sometimes, often, very often; range 0–3) [28]. In a previous cross-sectional study at age 3 years, we found that these teacher-rated subscales discriminated PAPA classified ADHD from non-ADHD significantly better than chance (*p* < 0.001) [24]. We used the dichotomized teacher symptom count where symptoms were scored as being not present (never/sometimes = 0) or present (often/very often = 1). In line with the ECI-4 manual, the cut-off score was set to the minimum number of symptoms necessary for the DSM-IV ADHD diagnosis; ≥ 6 on either the HI or the IA subscales [29]. Cronbach’s *α* was 0.91/0.90 for the HI/IA subscales. Pearson’s correlation between the two subscales was high (0.78; *p* < 0.001). Only about 56% of the 3-year sample received the ECI-4.

### At 8 years of age

#### Parents’ and teachers’ classifications by the Child Symptom Inventory-4 (CSI-4)

Parents and teachers responded to the CSI-4 (an equivalent inventory to the ECI-4), rated on a four-point Likert scale (never, sometimes, often, very often; range 0–3) [28]. We used the parent and teacher HI and IA subscales, each with nine items. Because the ECI-4 was only administered to 56% at age 3, we only had teacher reports at both 3 and 8 years for a subsample (*n* = 335).

We used the dichotomized symptom counts where symptoms were scored as being not present (never/sometimes = 0) or present (often/very often = 1). In line with the CSI-4 manual, the cut-off score was set to the minimum number of symptoms necessary for the DSM-IV ADHD diagnosis with ≥ 6 on either the HI or the IA subscales.

The Cronbach’s alphas were 0.88 for both parent HI and IA subscales, and 0.91 for the teacher HI and IA subscales. Pearson’s correlations between the two subscales were high for parents and teachers (both were 0.70; *p* < 0.001), while the parent–teacher intercorrelations were small to moderate (0.35–0.47).

About 66% percent of the parents of the participants at age 3 years completed the CSI-4 at child age 8 years. We therefore checked for differences between responders and non-responders. We found the responders slightly, but significantly better educated (mean = 14.94 years, SD = 2.21) than the non-responders (mean = 14.65 SD = 2.35, *t* = 2.03, *p* = 0.05). There were about the same proportions of boys among responders and non-responders (53% and 52%). There were no significant mean differences in the number of parent-reported ADHD symptoms at age 3 years, between responders and non-responders (mean = 3.97 SD = 3.84 vs. mean = 4.21 SD = 3.99; *p* = 0.32), nor in the number of teacher-reported ECI-4 ADHD symptoms (statistics not shown).

### Ethics

MoBa and the initial data collection was based on a license from the Norwegian Data Protection Agency and approval from The Regional Committees for Medical and Health Research Ethics. The MoBa cohort is currently regulated by the Norwegian Health Registry Act. The current study was approved by The Regional Committees for Medical and Health Research Ethics (2017/1276).

### Analytic plan

Internal consistencies were analysed using Cronbach’s *α*. We calculated the change in parent and teacher mean number of ADHD symptoms from age 3 to 8 years, using paired sample *t* tests, effect sizes by Cohen’s *d*, and correlations with Pearson’s *r*. In two separate multiple linear regression models, with age-8-year parent and teacher CSI-4 ADHD symptoms as two respective outcomes, we estimated the contributions of the age-3-year parent and teacher predictors (PAPA ADHD symptoms and ODD, teacher ECI-4 ADHD symptoms and sex). For the categorical analyses, we first calculated the proportion of children classified with ADHD at age 3 and/or 8 years by parents and teachers, respectively, and the agreement within informants across time by Cohen’s kappa (*κ*). We then estimated the stability of the HI, IA and combined presentations between timepoints, and the agreement between informants across time by calculating Cohen’s *κ*. Finally, we checked for sex differences, and whether the presence of ODD or lowering the HI and IA thresholds from 6 to 5 symptoms would improve stability.

## Results

The number of parent-rated ADHD symptoms dropped significantly from age 3 to 8 years, due to a significant reduction in the reported number of HI symptoms. There were no significant changes in the mean number of parent IA or teacher ADHD, HI or IA symptoms (Table 1).

**Table 1:** Mean number of symptoms and change from age 3 to 8 years

	*N*	3 years		8 years		Change		Effect sizes
		Mean	SD	Mean	SD	Mean	SD	Cohen’s *d*
Parents								
ADHD	783	3.96	3.84	2.58	4.05	− 1.38*	4.12	−0.33
HI	783	2.66	2.48	1.16	2.11	− 1.50*	2.44	−0.61
IA	783	1.30	1.79	1.41	2.28	0.11	2.34	0.05
Teachers								
ADHD	335	1.76	3.17	1.84	3.75	0.08	4.30	0.02
HI	335	0.97	1.90	0.83	2.05	− 0.14	2.42	−0.06
IA	335	0.79	1.59	1.01	2.05	0.22	2.32	0.09

*HI* hyperactivity/impulsivity, *IA* inattention

^*^p < .0005

There were moderate correlations between the parent-reported ADHD, HI and IA symptoms at age 3 and 8 years. For teachers, these correlations were low. Correlations between parent and teacher scores were low at 3 years, but considerably higher at 8 years (Table 2).

**Table 2:** Pearson’s correlations (*r*) between parent- (*n* = 783) and teacher-reported (*n* = 335) ADHD, hyperactivity-impulsivity (HI) and inattention (IA) symptoms at age 3 and 8 years

	1	2	3
ADHD symptoms			
1. Age 3 years parents (PAPA)	–		
2. Age 3 years teachers (ECI-4)	0.36***	–	
3. Age 8 years parents (CSI-4)	0.46***	0.18**	–
4. Age 8 years teachers (CSI-4)	0.27***	0.24***	0.53***
HI symptoms			
1. Age 3 years parents (PAPA)	–		
2. Age 3 years teachers (ECI-4)	0.33***	–	
3. Age 8 years parents (CSI-4)	0.44***	0.14*	–
4. Age 8 years teachers (CSI-4)	0.30***	0.25***	0.48***
IA symptoms			
1. Age 3 years parents (PAPA)	–		
2. Age 3 years teachers (ECI-4)	0.29***	–	
3. Age 8 years parents (CSI-4)	0.36***	0.19**	–
4. Age 8 years teachers (CSI-4)	0.18**	0.21***	0.51***

*PAPA* Preschool Age Psychiatric Assessment, *ECI-4* Early Childhood Inventory-4, *CSI-4* Child Symptom Inventory-4

***p < .0001; **p ≤ .001;*p < .05

The multiple linear regression model with age-8-year parent ADHD symptoms as outcome was overall statistically significant (*R*^2^ = 0.190, F (4, 388) = 22.75, *p* < 0.001), and only age-3-year parent ADHD symptoms contributed significantly (*β* = 0.43, *R*^2^ = 0.189, *p* < 0.001), while parent ODD, sex and teacher ADHD symptoms did not. The similar model with age-8-year teacher ADHD symptoms as outcome was also statistically significant (*R*^2^ = 0.129, F (4, 330) = 12.21, *p* < 0.001), age-3-year teacher ADHD symptoms explained 5.6% of the variance (*β* = 0.15, *R*^2^ = 0.056, *p* = 0.29), sex added about 4% (*β* = − 1.41, *p* < 0.001), and age-3-year parent ADHD symptoms an additional 3% (*β* = 0.19, *p* = 0.001), while ODD did not contribute significantly. The variance explained by sex, seemed related to teachers indicating a moderate increase in ADHD symptoms from age 3 to 8 years in boys, and a slight decrease in girls (statistics not shown).

**Fig. 1:**
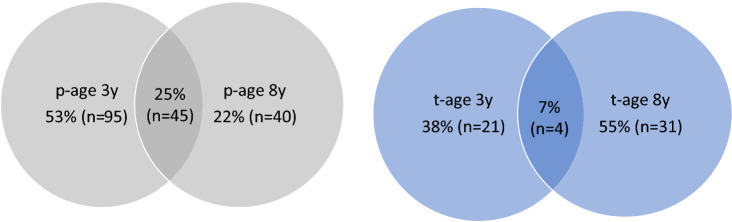
The proportions of pre-schoolers classified with Attention-deficit/hyperactivity disorder ADHD (either presentation) according to parents (*p*) (*n* = 180) and teachers (*t*) (*n* = 56) at age 3 and/or 8 years

With a categorical classification, 180 of 783 (23%) children met the symptom criteria for ADHD at age 3 and/or 8 years according to parents, and 56 of 335 (17%) according to teachers. Among these 180 and 56 children, only 25% and 7% met the criteria at both timepoints according to parents and teachers, respectively (Fig. 1).

The mean number of parent- and teacher-reported symptoms followed a clear pattern for each ADHD-category presented in Fig. 1, as well as for the children not categorized with ADHD at age 3 and/or 8 years. When the children were categorized with ADHD, their mean number of symptoms were much higher than the diagnostic thresholds, while when they were not categorized with ADHD, their mean number of symptoms were low (S1 and S2).

Table 3 shows the percentages of children classified with no ADHD or each of the three ADHD presentations (HI, IA and combined) at age 3 and 8 years. According to parents, HI was the most frequent presentation at age 3 years, while IA was most common at age 8 years, and the across-time-agreement was small [*κ* = 0.19 (95% CI = 0.13–0.25)]. Teachers indicated the similar number for HI and IA presentations at both ages, with only a slight across-time-agreement [*κ* = 0.06 (95% CI = 0.05–0.18)]. Table 3 shows some changes between the ADHD presentations, but the subgroups are mostly small. The only clear trend was the drop in HI presentations, where a total of 77/108 (71%) of the children classified with parent-reported HI presentation at age 3 years were no longer classified within any ADHD presentation at age 8 years.

**Table 3:** Percentages of children with hyperactivity/impulsivity (HI) presentation only, inattention (IA) presentation only, and combined presentations (≥ 6 symptoms of HI and IA) at age 3 and 8 years according to parents and teachers

Parents, *n* = 783	8 years, % (*n*)				
	None	HI only	IA only	Combined	Total
3 years % (*n*)					
None	77.0 (603)	1.4 (11)	2.6 (20)	1.1 (9)	82.1 (643)
HI only	9.8 (77)	0.6 (5)	1.1 (9)	2.2 (17)	13.8 (108)
IA only	0.3 (2)	0.0 (0)	0.3 (2)	0.0 (0)	0.5 (4)
Combined	2.0 (16)	0.3 (2)	0.5 (4)	0.8 (6)	3.6 (28)
Total	89.1 (698)	2.3 (18)	4.5 (35)	4.1 (32)	100.0 (783)
Teachers, *n* = 335					
3 years % (*n*)					
None	83.3 (279)	3.2 (11)	3.6 (12)	2.4 (8)	92.5 (310)
HI only	4.5 (15)	0.6 (2)	0.0 (0)	0.0 (0)	5.1 (17)
IA only	0.9 (3)	0.0 (0)	0.3 (1)	0.3 (1)	1.5 (5)
Combined	0.9 (3)	0.0 (0)	0.0 (0)	0.0 (0)	0.9 (3)
Total	89.5 (300)	3.9 (13)	3.9 (13)	2.7 (9)	100.0 (335)

At age 3 years, the ADHD presentations were classified by the Preschool Age Psychiatric Assessment interview with the parents, and the teacher presentations were classified by the Early Childhood Inventory-4. At age 8 years, parent and teacher ADHD presentations were classified by the Child Symptom Inventory-4

In the subsample with both parent and teacher reports (*n* = 328), the estimated parent–teacher agreement was small [*κ* = 0.22 (95% CI = 0.12–0.31)], but the parents and teachers agreed that 222 children were below the ADHD threshold at both time points. Only two children were above threshold according to both informants at both time points (Table 4).

**Table 4:** Number of children classified with ADHD at ages 3 and 8 years

	Parent ADHD				
	No	Age 3 years	Age 3 and 8 years	Age 8 years	Sum
Teacher ADHD					
No ADHD	222	34	8	11	275
Age 3 years	10	9	2	0	21
Age 3 and 8 years	0	1	2	1	4
Age 8 years	15	3	6	4	28
Sum	247	47	18	16	328

The categorical comparisons were done in the sample with parent and teacher reports (*n* = 335, 7 teacher reports were missing at age 8 years)

### Additional stability analyses

Sex or the presence of ODD at age 3 years did not significantly alter the proportion of children with HI and IA at age 3 or 8 years (statistics not shown). When lowering the thresholds for parent HI and IA (to 5 symptoms), we identified a few more children at both ages, but this did not improve the stability.

## Discussion

Our main findings were largely consistent with the four hypotheses. First, there was a significant reduction in the mean number of HI symptoms from age 3 to 8 years, but only for parents. For IA, there were similar mean numbers of symptoms at both timepoints, as hypothesized. Second, both parent- and teacher-reported ADHD symptoms at age 3 years significantly predicted ADHD symptoms reported by parents and teachers at age 8 years, respectively. Third, using categories, only 25% and 7% met the ADHD criteria at both timepoints according to parents and teachers, somewhat lower than hypothesized for parents. Also, in line with our hypothesis, we documented a large reduction in parent-reported HI presentation from preschool to school age, while teachers reported more similar rates at both ages. Fourth, there was very low parent–teacher agreement on ADHD classified above the diagnostic symptom thresholds, with only two children classified with ADHD according to both informants at both timepoints. Overall, our findings could suggest that age 3 years may be too early to apply the ADHD diagnostic criteria, especially if one requires parents and teachers to agree, in line with the recommendation from a previous study on young children [30].

Our finding of a considerable drop in the number of HI symptoms is in concordance with epidemiological studies of preschool children [9, 11], and a meta-analysis concluding that overall HI declines over time from the high levels seen during preschool [19], but in contrast to two population studies reporting a stable level to a slight decrease in HI symptoms during this period [30, 31].

For IA, we found low levels in the number of parent- and teacher-reported symptoms at age 3 years, in line with previous studies concluding that parent IA symptoms are rarely reported in pre-schoolers [11, 30, 32]. Previous studies with teacher reports have been less clear [33, 34], perhaps due to differences in child age between these studies. Despite the low preschool IA symptom level in the present study, there was no significant change in the mean number of parent- and teacher-reported symptoms from age 3 to 8 years.

Previous longitudinal clinical and high-risk studies have reported moderate to high ADHD stability from preschool to school age [2, 9, 10, 13, 15, 35]. We found moderate stability of parent-reported ADHD symptoms, and low stability for teachers. Also, we found modest to moderate contributions of age-3-years symptoms in the linear regression models with age-8-year symptoms as outcomes, and only 25% and 7% of children above thresholds at both timepoints, according to parent and teacher ratings, respectively. Our findings diverged from previous studies reporting that ≥ 70% of the pre-schoolers classified with ADHD were diagnosed again in school age [9, 10, 15]. However, these were clinical studies which likely explains their high stability, in line with the knowledge that few pre-schoolers are referred and treated for ADHD [36]. More in accordance with our findings, was one study that invited practitioners to refer children (age 3.5–5.5 years) with externalizing behaviour problems. They reported HI instability, with only 18% of the children with HI (≥ 6 symptoms) at three consecutive timepoints from preschool onwards [16]. For teachers, we found somewhat lower stability than one study reporting a stable high HI group of 11% according to teachers [33].

For the present study, caution is warranted as we only had teacher-reports in a sub-sample, resulting in few children classified within the subgroups. Still, the low levels of teacher-reported HI and IA are in accordance with previous studies concluding that parent reports on ADHD symptoms may be more useful than reports from teachers [20, 37], and one longitudinal study that found that only parent-reported ADHD symptoms significantly predicted an ADHD diagnosis at age 6 years [19]. Our finding of low stability in the number of teacher-reported ADHD symptoms, could suggest that these symptoms may be more difficult to detect in a preschool setting, compared to in school where there are higher expectations to child behaviour. Also, the stability of teacher-reported symptoms may be additionally reduced by different teachers being informants at the two timepoints.

In our study, both parents and teachers reported that a small percentage of children changed from HI at 3 years to IA at 8 years. This seems to be in line with the results from a population study following symptom trajectories concluding that the frequency of HI symptoms tended to slightly decrease with age, whereas the frequency of IA symptoms substantially increased to the age of 6 years [31]. We also found some children above threshold only at age 8 years, in line with a previous study noting substantial fluctuation in diagnostic profiles from age 4–5 to 6–7 years, with 16% losing the ADHD diagnosis and 34% new cases at the last timepoint [11].

Obtaining information from both parents and teachers is recommended to ascertain that the ADHD diagnostic thresholds are present in at least two settings. Our results could suggest that this may be too strict in early preschool years, in line with studies showing low parent–teacher agreement [14, 19, 30]. Still, parent-reported age-3-year ADHD symptoms added a little to that of age-3-year teacher ADHD symptoms in the regression model where age-8-year teacher ADHD symptoms was outcome. Also in this model, sex added to the variance (4%), indicating that teachers may be poor at noticing ADHD symptoms in girls, in line with our previous study [20]. The low parent–teacher agreement was most clearly demonstrated when we found only two children identified by both parents and teachers at both timepoints, almost identically low to the previously mentioned German study [14]. However, it should be noted that within the teacher-subsample, there were too few children classified with ADHD (at one or both time points by either informant), to make firm conclusions. Also, the use of categories may have artificially decreased agreement somewhat.

### Additional stability analyses

Neither controlling for sex or ODD, the most common co-occurring disorder to ADHD, or lowering the HI and IA thresholds altered the stability of classified ADHD significantly from age 3 to 8 years.

### Strengths and limitations

Our study has important strengths in the population-based cohort design and the use of a parent diagnostic interview when the children were 3 years, but it also has limitations. There were selection biases due to attrition in MoBa and the ADHD-study [21, 38], and a considerable attrition from age 3 to 8 years. However, we found only slight differences between responders and non-responders in mean parent education, with no differences in sex distribution or in the number of parent- and teacher-reported ADHD symptoms at age 3 years. Together this indicates that attrition at 8 years should not have biased our results substantially. Also, a previous MoBa-study reporting on ADHD found differences between participants and the general population to be small and assumed limited effects on generalizability [39]. To estimate the accuracy of the sampling procedure at age 3 years, we checked and found that among the recruited MoBa controls, only six children were later classified with ADHD according to PAPA and one child according to teacher ECI-4 at age 3½ years. Before recruitment to our study, another MoBa sub study on autism spectrum disorder (ASD) recruited children, limiting the likelihood of ASD within our sample, although we cannot rule out that children with HI and/or IA at age 3 years, may have other neurodevelopmental problems at age 8 years. We checked whether ODD, the most common co-occurring disorder to ADHD affected stability and found that it did not. The large reduction in parent-reported HI, may indicate an overweight of concerned parents being recruited at age 3, perhaps simply concerned for age-appropriate high activity levels. At age 8 years, we unfortunately only had available parent and teacher questionnaire ratings of ADHD symptoms, not equivalent to ADHD diagnosis which includes a broader evaluation (impairment, language, cognition, etc.). The lack of conformity in measures in our study, may have increased the likelihood of misclassifications. However, even population studies using psychiatric diagnostic interviews both in preschool and school age show lack of stability, with broad confidence intervals in risk estimates for ADHD [2, 13]. Also, another population study reporting all psychiatric disorders diagnosed at age 4 and 6 years, found that only 1.8% of the children (17/86) had diagnoses at both timepoints [40]. Only having two timepoints, makes the present study susceptible to measurement errors (random variation, regression to the mean). Finally, we only had teacher ECI-4 at age 3 for a subsample, reducing statistical power.

## Conclusions and future directions

Our findings from this population study, could suggest that age 3 years may be too early to apply the ADHD diagnostic symptom criteria, especially if one requires parent–teacher agreement. The reduction in parent-reported HI symptoms was large from preschool to school age. Therefore, clinicians should exercise caution in communicating concern about HI symptoms in preschool children, even when this information is provided in a diagnostic interview. The low reports of ADHD symptoms by teachers and low stability from age 3 to 8 years, may be related to preschool teachers perhaps not being trained in observing symptoms. However, the utility of preschool teacher-reported ADHD ratings should be investigated in a clinical study. Future studies should consider educating participating teachers on ADHD symptoms and investigate whether this will increase predictive value of their ratings, and the agreement between informants. Considering the void in the literature, with only three previous studies examining the validity of HI and IA symptoms in preschool children [11, 30, 32], future studies should examine which combinations and numbers of preschool parent- and teacher-reported HI and/or IA symptoms best predict ADHD in school age.

### Supplementary Information

Below is the link to the electronic supplementary material.

Supplementary file1 (DOCX 15 KB)
