# Supplementary material for: Attention-deficit/hyperactivity disorder from preschool to school age: change and stability of parent and teacher reports
Source: Eur Child Adolesc Psychiatry. 2022 Jun 23;32(10):1947–55. doi: 10.1007/s00787-022-02019-1 (PMC10533600; doi:10.1007/s00787-022-02019-1)
Supplement: Supplementary file 1 — Supplementary file1 (DOCX 15 KB) [file 787_2022_2019_MOESM1_ESM.docx]

**Table S1** Mean number of parent ADHD symptom at ages 3 and 8 years for children classified with/without ADHD (above/below diagnostic thresholds) according to parents at these timepoints.

|  |  | 3-years | | 8-years | | Change | |
| --- | --- | --- | --- | --- | --- | --- | --- |
|  | N | Mean | S.D. | Mean | S.D. | Mean | S.D. |
| No ADHD at age 3 or 8 years | 603 | 2.48 | 2.41 | 1.26 | 2.08 | - 1.22 | 2.69 |
| ADHD at age 3 years only | 95 | 9.75 | 2.61 | 2.30 | 2.39 | - 7.45 | 3.19 |
| ADHD at ages 3 and 8 years | 45 | 10.83 | 3.37 | 12.98 | 3.04 | +2.16 | 4.46 |
| ADHD at age 8 years only | 40 | 4.78 | 2.55 | 11.30 | 2.71 | +6.53 | 3.60 |

**Table S2** Mean number of teacher ADHD symptoms at ages 3 and 8 years for children classified with/without ADHD (above/below diagnostic thresholds) according to teachers at these timepoints.

|  |  | 3-years | | 8-years | | Change | |
| --- | --- | --- | --- | --- | --- | --- | --- |
|  | N | Mean | S.D. | Mean | S.D. | Mean | S.D. |
| No ADHD at age 3 or 8 years | 279 | 0.87 | 1.66 | 0.73 | 1.73 | - 0.14 | 2.18 |
| ADHD at age 3 years only | 21 | 10.48 | 2.86 | 1.24 | 2.41 | - 9.23 | 3.96 |
| ADHD at ages 3 and 8 years | 4 | 10.50 | 1.91 | 10.50 | 5.07 | 0.00 | 4.69 |
| ADHD at age 8 years only | 31 | 2.71 | 3.06 | 11.15 | 3.30 | +8.44 | 4.02 |
